# Mr. Westcote's Reasons for Deferring Amputation

**Published:** 1804-01-01

**Authors:** J. P. Westcote

**Affiliations:** Surgeon. Martock


### *To the Editors of the Medical and Phyſical Journal.*

GENTLEMEN,

AS improvement in the different branches of Medicine, is the grand object of your very valuable Journal, I conceive it to be the duty of every professional man to record every case which may occur to him, that appears likely in any measure to lead to the public good, and particularly any circumstance which may happen to prevent, and may occasionally obviate, the necessity of a dreadful operation in Surgery, where not only the loss of a limb, but sometimes the loss of life is the consequence.

In taking notice of the following case I do not pretend to propose any new practice, more than might occur to every judicious surgeon; but the recording such a case may make an impression on the minds of young practitioners, who are sometimes intimidated at meeting with a violent hæmorrhage, and may be the means of a similar plan plan being adopted, to the saving of many limbs if not lives.

In the beginning of September, 1802, Rose Clothier, aged about fifty, of an athletic habit, received a wound in the upper part of her left foot, from a reaping hook inflicted by a person who was standing next to her reaping, striking off an ear of corn, the hook being reversed; the wound was consequently in a diagonal direction, penetrated between the metatarsal bones of the third and fourth toes, and wounded the artery, from whence there was a considerable hæmorrhage.

The accident happened early in the morning, I think about eight o'clock. The medical gentlemen who were in attendance, found every effort frustrated which they employed for several hours to stop the hæmorrhage, and proposed amputation as the only resource for the preservation of life.

I was called in about six hours after the accident, and well knowing the impracticability of coming to tye an artery situated under the metatarsal bones and among the flexor tendons of the foot, I did not hesitate what to do as the most likely means to preserve the limb. The grand object being to retard the velocity of the circulation to that extremity, so as not to endanger the life of the limb, and thereby give an opportunity for a styptic to take a due effect; and I had the extreme felicity of witnessing the propriety of my conclusion.

I applied a tourniquet above the knee to the popliteal artery, and kept it to that degree of pressure which did not compleatly stop the circulation, but so far impeded it that after I had applied a small tent of lint dipped in colcoth. vit. and the necessary superficial dressings over, I did not perceive the least hæmorrhage. These dressings were permitted to remain in statu quo for three days, and the tourniquet was gently loosened every day. When the dressings were removed on the third day no hæmorrhage ensued, though the tourniquet was completely loose; the tent was gradually discharged, and the wound healed in a short time.

This case particularly shews the propriety of a deliberate and cool determination on the necessity of amputation; we ought in all cases, where the life of the patient is not immediately endangered, to give every means a trial, as we can but have recourse to the only alternative at last.

*Martock,* I am, &c.

*December* 3, 1803. J. P. WESTCOTE, Surgeon.

